# Methods for estimating disease transmission rates: Evaluating the precision of Poisson regression and two novel methods

**DOI:** 10.1038/s41598-017-09209-x

**Published:** 2017-08-25

**Authors:** Carsten Kirkeby, Tariq Halasa, Maya Gussmann, Nils Toft, Kaare Græsbøll

**Affiliations:** National Veterinary Institute, Technical University of Denmark, Bülowsvej 27, DK-1870 Frederiksberg C, Denmark

## Abstract

Precise estimates of disease transmission rates are critical for epidemiological simulation models. Most often these rates must be estimated from longitudinal field data, which are costly and time-consuming to conduct. Consequently, measures to reduce cost like increased sampling intervals or subsampling of the population are implemented. To assess the impact of such measures we implement two different SIS models to simulate disease transmission: A simple closed population model and a realistic dairy herd including population dynamics. We analyze the accuracy of different methods for estimating the transmission rate. We use data from the two simulation models and vary the sampling intervals and the size of the population sampled. We devise two new methods to determine transmission rate, and compare these to the frequently used Poisson regression method in both epidemic and endemic situations. For most tested scenarios these new methods perform similar or better than Poisson regression, especially in the case of long sampling intervals. We conclude that transmission rate estimates are easily biased, which is important to take into account when using these rates in simulation models.

## Introduction

Simulation models are widely used to model spread and control of many different infectious diseases in both human and veterinary medicine^1^, for instance malaria^2^, SARS^3^, STDs^4^, influenza^5^, MRSA^6^, Ebola^7^, rabies^8^, scrapie^9^ and mastitis^10^. In these models, transmission rates are used to describe the flow of individuals in a population going from a susceptible state to an infected state, and it is important to obtain a realistic estimate of the transmission rates in order to create a useful and realistic simulation models for decision support^11–13^. Accurate estimation of this rate is important because it can have a major influence on model predictions and conclusions (e.g. refs^11,14^). The rate may have different names depending on the specific mathematical representation of transfer between susceptible and disease states, e.g. infection rate, transmission coefficient and transmission rate. This key parameter is difficult to estimate for most host-pathogen models because natural processes are stochastic, and transmission events are influenced by other parameters than what can be included in a transmission model^1,15^. Thus, large datasets are often needed to reach a good estimate.

Experiments that can be used to estimate the transmission rate are often both difficult and time-consuming to conduct^12^. A means to obtain longitudinal data for estimation of the transmission rate can be to sample a subpopulation instead of the whole population, as for instance the study of Backer *et al*.^16^ where field data were used to estimate transmission rate of hepatitis E virus in pigs, subsampling down to 5% of the population. This can be the case for instance if animals must be caught prior to sampling, if the population is large, or if the test for disease is expensive^17^. In such situations subsampling can be a convenient method, but the precision of the estimate can be jeopardized by sampling fewer individuals. We wanted to quantify this in the present study.

In longitudinal studies of disease transmission, a short sampling interval is often desired because it provides precise data on the incidence. Ideally, the period between sampling points should be the average time between the infection of one individual and the infection of the next individual that is infected from the first individual^18^. However, it is often not practical to sample with very short sampling intervals. For instance, Caley and Ramsey^12^ used a sampling scheme with three samplings per year, and Zadoks *et al*.^11^ sampled every three weeks. If the time between sampling points is long, some individuals may be infected and recovered between sampling points and therefore not recorded. Hence, the transmission rate will be underestimated. On top of that, data from biological systems most often contains noise that may impact the estimated rate. Numerous studies have estimated the transmission rate from field data^11,16,19^. However, few studies have evaluated how precise the estimated transmission rates actually are. O’Dea *et al*.^20^ recently used Poisson regression to estimate the transmission rate and showed that the precision of the estimate increased with the number of outbreaks used for estimation. Cook *et al*.^21^ used Bayesian inference to show that it is important to take spatial population heterogeneity into account when estimating transmission rates. To our knowledge, no previous studies have explored the precision of the estimated transmission rate in relation to a known simulated transmission rate, the sampling interval and the subsampling percentage.

In the present study we wanted to estimate the precision of transmission rates estimated from population data, and chose a dairy herd as the study population. Modern dairy cattle herds constitute a good example of a population where many data are available on the individual level. Furthermore, they represent a controlled system mostly with free-stall systems that invoke homogeneous mixing between the lactating cows. These facts, combined with a fair number of diseases that can be present in a dairy herd, makes it a good study unit for estimating disease transmission rates.

A few studies have recently been conducted to estimate the transmission parameter from data in cattle herds considering infections with salmonella^18^ and mastitis^11,19,22–24^. Most of these studies have used Poisson regression to estimate the transmission rate from real life data, although a few also used negative binomial regression. A challenge of estimating transmission rates is that a low rate can result in very low numbers of or no new infections per time period, which can prevent the use of regression procedures with a log-link. We therefore developed two new methods to estimate the transmission rate, which are robust to these challenges.

The advantage of simulated data is that we can measure exactly how precise the estimated transmission rates are. In this study we wanted to use two models with known transmission rate, a simple SIS model (SISsim) with only two disease states (Susceptible and Infected), and a complex SIS model (SIScom) with three disease states (Susceptible, Infected - subclinical, and Infected - clinical) reflecting a realistic system where external factors such as death and treatments of infected individuals influence the disease dynamics within the system.

The objectives of this study were to explore the effect of sampling interval, subsampling of the population and the size of the transmission rate itself, on the estimated transmission rate. Furthermore, we wanted to assess the precision and robustness of the Poisson regression to extract disease transmission rates, because this method is often used. We also present two new methods for estimating the transmission rate that are simpler and more robust. These methods have, to our knowledge, not been used to estimate transmission rates before. We compare the three methods to assess which method estimate the transmission rate to be closest to the known transmission rate.

## Methods

**Figure 1:**
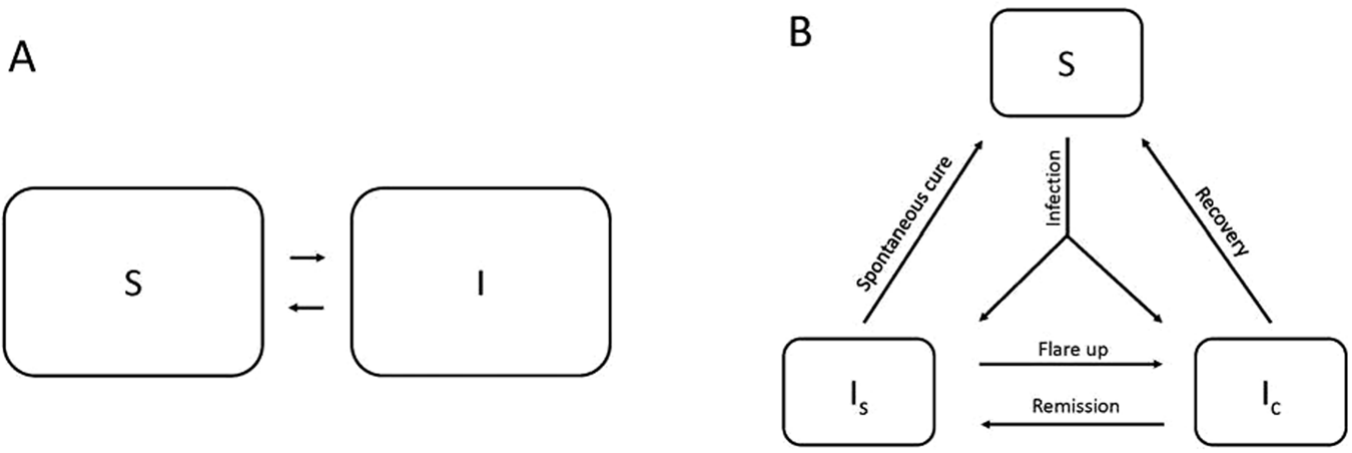
The transmission frameworks in the SISsim (**A**) and the SIScom (**B**) models. *S* = Susceptible, *I* = Infected, *I*_*S*_ = Infected and subclinical, *I*_*C*_ = Infected and clinical. The transmission parameters for (**B**) are explained in Table 1 in the supplementary material.

All simulations were run in R^25^. We used two stochastic discrete-time simulation models to model disease spread, namely a SIS model (SISsim) and a SIScom model, described below (see Fig. 1). We simulated contagious frequency-dependent transmission of a disease in daily time steps in both models^1,26^. This approach allowed us to compare a simple model (SISsim) with a model with natural noise (SIScom).

We combined the two simulation models (SISsim and SIScom) with two situations, namely an epidemic and an endemic situation, resulting in four different scenarios. These four scenarios were analyzed with three different methods: Poisson regression and two new methods (described below). Using these methods, we varied the transmission rate, the sampling interval and the subsampling fraction of the population. For all simulations the time span sampled was one year, so if sampling interval was one day there would be 365 data points, but if sampling interval was 30 days there would only be 12 data points per simulation. The three different methods were given the same data points to estimate the transmission rates.

The new methods rest on an assumption of the simulated system being in equilibrium. However, the derivations show that the bias of the equilibrium assumption is sometimes of a smaller order compared to the overall estimate. To estimate the bias introduced by this assumption, we chose to simulate both epidemic and endemic situations of disease. In comparison, the Poisson regression relies on an estimate of average number of infected and susceptible units between sampling times, which is not the case for the new methods. Thus the Poisson method introduces another type of bias.

### SISsim simulation model

The first model was a SIS model (SISsim) with a population with 1000 individuals^26^. The individuals in the SISsim models had no extra attributes (e.g. age, pregnancy, production status, etc.). We assumed homogenous mixing of individuals with the probability of infection being proportional to the fraction of infected individuals in the population. The method is analogue to the system of differential equations:

1 $$\frac{dS}{dt}=-\,\beta \frac{SI}{N}+\alpha I$$

2 $$\frac{dI}{dt}=\beta \frac{SI}{N}-\alpha I$$

where *β* is the disease transmission coefficient (transmission rate), *α* is the recovery rate, *S* is the number of susceptible individuals, *I* is the number of infected individuals and *N* is the total number of individuals. The rates *α* and *β* are given in Table 1 in the supplementary material. However, we model in discrete time steps of one day therefore the daily probability of infection, *P*, for each susceptible individual^1,27^ becomes:

3 $$P=1-\exp (\,-\,\beta I/N)$$

whether an individual would become infectious was decided by daily Bernoulli draws using *P* as probability. Data collection ran for 365 days and the number of individuals in S, I and N were recorded for each time step, as well as the number of newly infected individuals. We repeated the simulations 50 times to increase the robustness of the estimated rates.

We then simulated disease testing in intervals, mimicking a field study where sampling is conducted in different time intervals.

### SIScom simulation model

We also created a realistic SIS model (SIScom) to test how the methods would perform in a realistic setting with fluctuating population dynamics. The SIScom model is a complete individual based model of a cow herd, were cows can be culled based on low milk yield or high somatic cell count, and can befall ill and slaughtered, there is also separation between cows in different production stages (lactating, drying off, giving birth). The SIScom model was created by merging the transmission framework for intramammary infection created by Halasa *et al*.^14^ with the iCull simulation model described by Kirkeby *et al*.^28^. Briefly, the iCull model simulates a dairy herd with 200 cows and after a 3 year burn in period the SIScom model is started. We recorded data during a single simulated year. In the model, all cows are simulated realistically where they typically calve and lactate for around 350 days. Then they are dried off in about 56 days and then they calve again. The farmer takes weekly decisions on culling, choosing the lowest producing cows for slaughter. Individuals can also be culled due to diseases like infection. Individuals are only culled when new heifers, raised in the herd, enter the lactation stage. Thus the number of individuals is kept fairly stable. The transmission framework was adopted from Halasa *et al*.^14^, simulating intramammary infections (IMI). Susceptible individuals can be infected with IMI and enter a subclinical or a clinical stage. Infections in subclinical individuals can flare up, and the infection in clinical individuals can be subclinical again (remission). Finally, infected individuals can recover from disease and be susceptible again. The disease spread is analogue to the differential equations:

4 $$\frac{dS}{dt}=-\,\beta \frac{{I}_{S}+{I}_{C}}{N}S+{\alpha }_{S}{I}_{S}+\eta \,{\alpha }_{C}{I}_{C}-\mu S+\mu N$$

5 $$\frac{d{I}_{S}}{dt}=\rho \beta \frac{{I}_{S}+{I}_{C}}{N}S-{\alpha }_{S}{I}_{S}+\mathrm{(1}-\eta )\,{\alpha }_{C}{I}_{C}-{\gamma }_{S}{I}_{S}-\mu {I}_{S}$$

6 $$\frac{d{I}_{C}}{dt}=\mathrm{(1}-\rho )\beta \frac{{I}_{S}+{I}_{C}}{N}S-{\alpha }_{C}{I}_{C}+{\gamma }_{S}{I}_{S}-\mu {I}_{C}$$

where *S* is the number of susceptible individuals, *I*_*S*_ is the number of subclinically infected individuals, *I*_*C*_ is the number of clinically infected individuals, and any other parameters are given in Table 1 in the supplementary material. Infections occur on quarter level, independently of other quarters on the same cow. Hence, there are essentially 800 units exposed to infection. The initial prevalence in the model was 10% on quarter level in the epidemic scenarios. We ran 30 simulations in the SIScom model framework which was deemed enough to reach convergence (data not shown).

Individuals entering the clinical state are given a fixed duration of three days and are then healed, going to the susceptible state, or remission can occur so they go to the subclinical state again.

In the both models we know the true status of each individual, thus leaving out noise from the test procedure.

### Subsampling procedure

In the SISsim model, the population subsampling was performed by initially and randomly choosing a given number of individuals for testing at each test day. These individuals were followed over the simulations. Similarly, in the SIScom model we first randomly chose a given number of individuals for testing. If these individuals were in dry off on a given test day, and therefore did not have a test result, they were not included in the test day recording. After dry off they were included again. If a cow in the subsample population was culled, it was replaced by another cow. Replacement cows were the youngest lactating cows, which have a higher survival rate than older cows. We ran different sampling scenarios in which 100%, 75%, 50% or 25% of the population was sampled.

### Estimation methods

We used three different methods to estimate the transmission parameters: Poisson regression and two new methods.

#### Poisson

The poisson regression method estimates the transmission rate using Poisson regression with a log-link as described in Zadoks *et al*.^11^:

7 $$\widehat{\mathrm{log}({I}_{N})}=\,\mathrm{log}\,(\beta )+\,\mathrm{log}\,(\frac{{S}_{int}{I}_{int}}{{N}_{int}})$$

where $\widehat{\mathrm{log}({I}_{N})}$ is the expected log number of new infections per sampling interval. *S*_*int*_ is the number of susceptible individuals in each sampling interval multiplied with the sampling interval, plus half of the new infections per sampling interval multiplied with the sampling interval. Thus, *S*_*int*_ is the estimated number of individual days at risk based on the average number of susceptible in the sampling period. *I*_*int*_ is the number of continuously infected individuals in each sampling interval multiplied with the sampling interval, plus half of the new infections per sampling interval multiplied with the sampling interval. *N*_*int*_ is the total number of individuals in each sampling interval multiplied with the sampling interval. The term log (*S*_*int*_*I*_*int*_/*N*_*int*_) was used as an offset. Using the offset we fix the slope of the regression to 1, thereby obtaining the transmission rate, *β*. In the SIScom model, the same Poisson model was used units were not cows but quarters (teats).

Finding *S*_*int*_ and *I*_*int*_ from field studies so they can be used in a Poisson regression analysis can be complicated given that there can be many introductions and exits of animals between samplings. Therefore we derived two other methods for estimating the transmission rate, which use only the number of animals in the different categories (*I*_*N*_, *I*, *N*, and *S*).

#### Method 1

The estimate for the transmission rate in Method 1 is given by:

8 $$\beta =\frac{-\,\mathrm{log}\,\mathrm{(1}-{I}_{N}/I)}{T\,S/N}$$

where *β* is the mean estimated transmission rate, *I*_*N*_ is the number of new infections since the previous sampling, *I* is the number of infected individuals, *S* is the number of susceptible individuals, and *T* is the sampling interval.

#### Method 2

The estimate for the transmission rate in Method 2 is given by:

9 $$\beta =-\,\frac{1}{T}\,\mathrm{log}\,(1-{I}_{N}(\frac{1}{I}+\frac{1}{S}))$$

with the same parameters as described for Method 1.

#### Derivations

Both new methods are based on the assumption that the system is in equilibrium (endemic state), however, we shall show that this assumption introduces bias that is often small. First we note that the equations describing the SIScom model can be rewritten to:

10 $$\begin{array}{rcl}\frac{dS}{dt} & = & -\,\beta \,\mathrm{(1}+\delta )\frac{{I}_{C}S}{N}+{\alpha }_{S}\delta {I}_{C}+\eta {\alpha }_{C}{I}_{C}+\mu \mathrm{(1}+\delta ){I}_{C}\end{array}$$

11 $$\begin{array}{ccc}\, & = & -\,\tilde{\beta }\frac{{I}_{C}S}{N}+\tilde{\alpha }{I}_{C}\end{array}$$

where *δ* = *I*_*S*_/*I*_*C*_, $\tilde{\beta }=\beta \mathrm{(1}+\delta )$, and $\tilde{\alpha }={\alpha }_{S}\delta +\eta {\alpha }_{C}+\mu \mathrm{(1}+\delta )$ under the assumption that we are at the equilibrium, so that *δ* is a constant. This amounts to reducing the system to two processes going only between the susceptible and infectious individuals. For the remainder of the manuscript *α* and *β* can thus be thought of either as the original *α* and *β* from the SISsim model or $\tilde{\alpha }$ and $\tilde{\beta }$ from the SIScom model.

Equilibrium is defined as *dS*/*dt* = 0, and therefore:

12 $$\frac{S}{N}=\frac{\alpha }{\beta }$$

when in equilibrium there will still be new infections as some are replaced and some recover which is included in the *α* parameter. Therefore, the number of new infections can be described as:

13 $$\frac{d{I}_{N}}{dt}=\beta \frac{IS}{N}-\alpha {I}_{N}$$

Being in equilibrium *S* and *I* are constants and the solution to the differential equation is:

14 $$\begin{array}{rcl}{I}_{N}\,(t) & = & \frac{\beta }{\alpha }\frac{IS}{N}+c\,\exp \,(\,-\,\alpha t)\end{array}$$

15 $$\begin{array}{rcl}\, & = & I\mathrm{(1}-\exp \,(\,-\,\alpha t))\end{array}$$

using the properties of the equilibrium as described by equation (12), and setting *c*, such that *I*_*N*_(0) = 0 and *I*_*N*_(∞) = *I*. Solving for *α*, where an explicit sampling time interval *t* = *T* is used:

16 $$\alpha =-\,\frac{\mathrm{log}\,\mathrm{(1}-{I}_{N}/I)}{T}$$

Inserting this into eq. (12) and rearranging, gives method 1, eq. 8:

$$\beta =\frac{-\,\mathrm{log}\,\mathrm{(1}-{I}_{N}/I)}{T\,S/N}$$

Method 1 functions if all new infections are observed. However, this is not always the case when sampling in reality. Especially, individuals that recover and become reinfected should be counted as new infections, which is most often not possible. Therefore, a second method that only counts individuals susceptible at the previous sampling as new infections was developed:

17 $$\frac{d{I}_{N}}{dt}=\beta \frac{I(S-{I}_{N})}{N}-\alpha {I}_{N}$$

Again being in equilibrium *S* and *I* are constants and the solution to this differential equation is:

18 $$\begin{array}{rcl}{I}_{N}(t) & = & \frac{\frac{\beta IS}{N}}{\frac{\beta I}{N}+\alpha }+c\,\exp \,(\,-\,(\alpha +\beta I/N)t)\\  & = & (\frac{1}{{S}^{-1}+{I}^{-1}})\,(1-\exp \,(\,-\,\alpha \mathrm{(1}+I/S)t))\end{array}$$

and isolating for *α*:

19 $$\alpha =-\,\frac{\mathrm{log}\,(1-{I}_{N}\,({S}^{-1}+{I}^{-1}))}{T\mathrm{(1}+I/S)}$$

Inserting this into eq. (12) and rearranging while noting that *S*/*N*(1 + *I*/*S*) = 1, gives method 2, eq. 9:

$$\beta =-\,\frac{1}{T}\,\mathrm{log}\,(1-{I}_{N}(\frac{1}{I}+\frac{1}{S}))$$

Note that *α* for both methods describes a combined recovery and replacement rate. The *α* derived is not the recovery rate and can be heavily biased if the system is out of equilibrium.

#### Bias

Both new methods rely on the assumption of the disease being in equilibrium, but in reality this state is practically impossible to obtain due to population dynamics and stochastic events. In a simulation model, there are also many factors that drives a system away from equilibrium, e.g. the fact that we are counting integer number of individuals in principle prohibits the system from being in exact equilibrium if the ratio of *α*/*β* is not precisely so that it could be *S*/*N* for integers. To estimate this error, let *ε* represent the bias so that *εS* is the observed number of susceptible, where *S* is the number of susceptible in equilibrium. The observed number of susceptible is substituted into eq. (14) so that it becomes:

20 $${I}_{N}(t)=\frac{I}{\varepsilon }\mathrm{(1}-\exp \,(\,-\,\alpha t))$$

and inserting into the estimate for *β*:

21 $$\beta =\frac{-\,\mathrm{log}\,\mathrm{(1}-\varepsilon {I}_{N}/I)}{T\varepsilon S/N}\approx \frac{\varepsilon {I}_{N}/I}{T\varepsilon S/N}-\frac{{(\varepsilon {I}_{N}/I)}^{2}}{2T\varepsilon S/N}+\cdots =\frac{{I}_{N}/I}{T\,S/N}-\frac{\varepsilon {({I}_{N}/I)}^{2}}{2T\,S/N}+\cdots $$

where the local approximation is done by Taylor expansion. Notice that the bias disappears in the first order term.

A similar exercise can be done for method 2, where the bias does not disappear.

$$\beta =-\,\frac{\mathrm{log}\,(1-{I}_{N}(\frac{1}{I}+\frac{1}{\varepsilon S}))}{T\frac{\varepsilon SI}{N}(\frac{1}{I}+\frac{1}{\varepsilon S})}\approx \frac{{I}_{N}/I}{T\varepsilon S/N}-\frac{{I}_{N}^{2}\,(\frac{1}{I}+\frac{1}{\varepsilon S})/I}{2T\varepsilon S/N}+\cdots $$

### Simulated data

The two new methods derived above are theoretically for use primarily in an equilibrium situation. However, data is not always obtained during equilibrium. Therefore we simulated both epidemic and endemic scenarios with both simulation models to estimate the error made by the methods.

The epidemic scenarios were simulated by initially infecting 10 of 1000 individuals in the SISsim model, and 10 of 800 quarters (units) in the SIScom model. The endemic scenarios were simulated in both models by starting in equilibrium. After estimating the transmission rates from both data sets, we calculated the variation in the estimate over the number of simulations, using 2 to 50 random samples from the SISsim model data and 2 to 30 samples from the SIScom model data. We divided the estimated rate by the simulated rate to obtain a precision measure, and then took the standard deviation of this number. We then evaluated if the model had converged.

### Data availability statement

The data sets used can be acquired from the corresponding author upon request.

## Comparison of Methods

We compared the performance of the three models in all modelled scenarios and combinations of test interval and transmission rate. For each situation we found the best method, which was the one that estimated the transmission rate to be closest to the known true rate. Then we calculated if the mean estimate of the other two methods overlapped with *mean* ± 1.96 · *sd* of the best method. If this was the case, there was no significant difference between the methods.

## Results

In the SISsim model, 50 repetitions were enough to reach convergence, and in the SIScom model, 30 repetitions were enough to reach convergence (data not shown).

**Figure 2:**
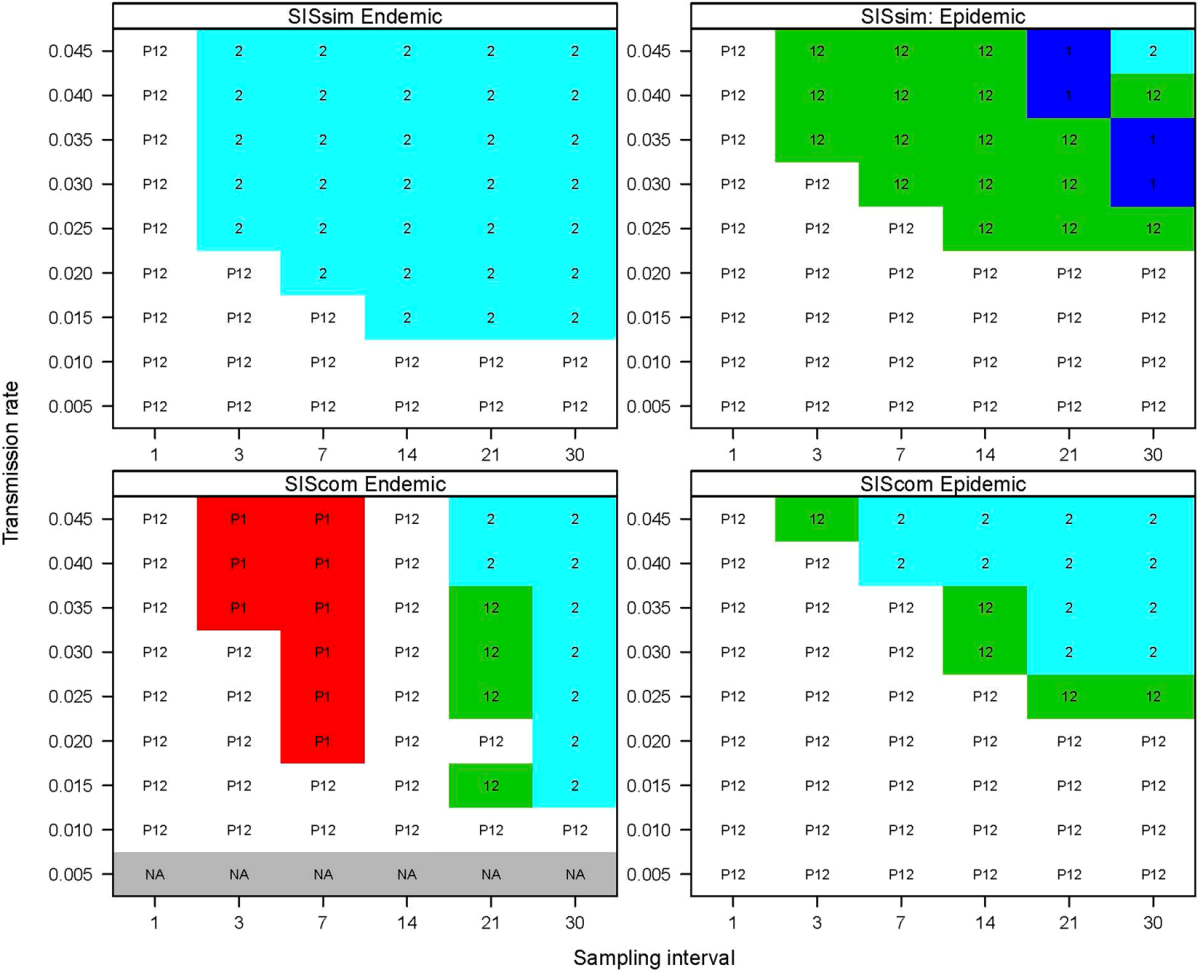
The best methods for each scenario and combinations of transmission rate and test interval. *P* = Poisson regression, 1 = Method 1, 2 = Method 2. In cases where more than one method is indicated, no significant difference was found between the methods. Colors are specific to each combination.

The best methods for each scenario and combinations of transmission rate and sampling interval are shown in Fig. 2. In all four scenarios, all three methods perform equally when the sampling interval is large and transmission rate is low, and also when the transmission rate is high and the sampling interval small (Fig. 2). For the SISsim model in the endemic scenario, Method 2 was significantly better than the other two. For the same model with the epidemic scenario, model 1 and 2 performed equally well and significantly better than the Poisson method, but they alternate when the transmission rate and the test interval are high (Fig. 2).

For the SIScom model in the endemic scenario, the Poisson method and Method 1 performed significantly better than Method 2 when the sampling interval is 3 or 7 days and the transmission rate is high. When the sampling interval was 14 days, all three methods performed equally. At larger sampling intervals Method 1 and Method 2 performed better than Poisson. Likewise, for the SIScom model in the epidemic scenario, Method 1 and Method 2 performed better for large intervals and high transmission rates. For the SIScom model, Method 2 generally performed better than the other two at large sampling intervals and transmission rates.

### SISsim model

**Figure 3:**
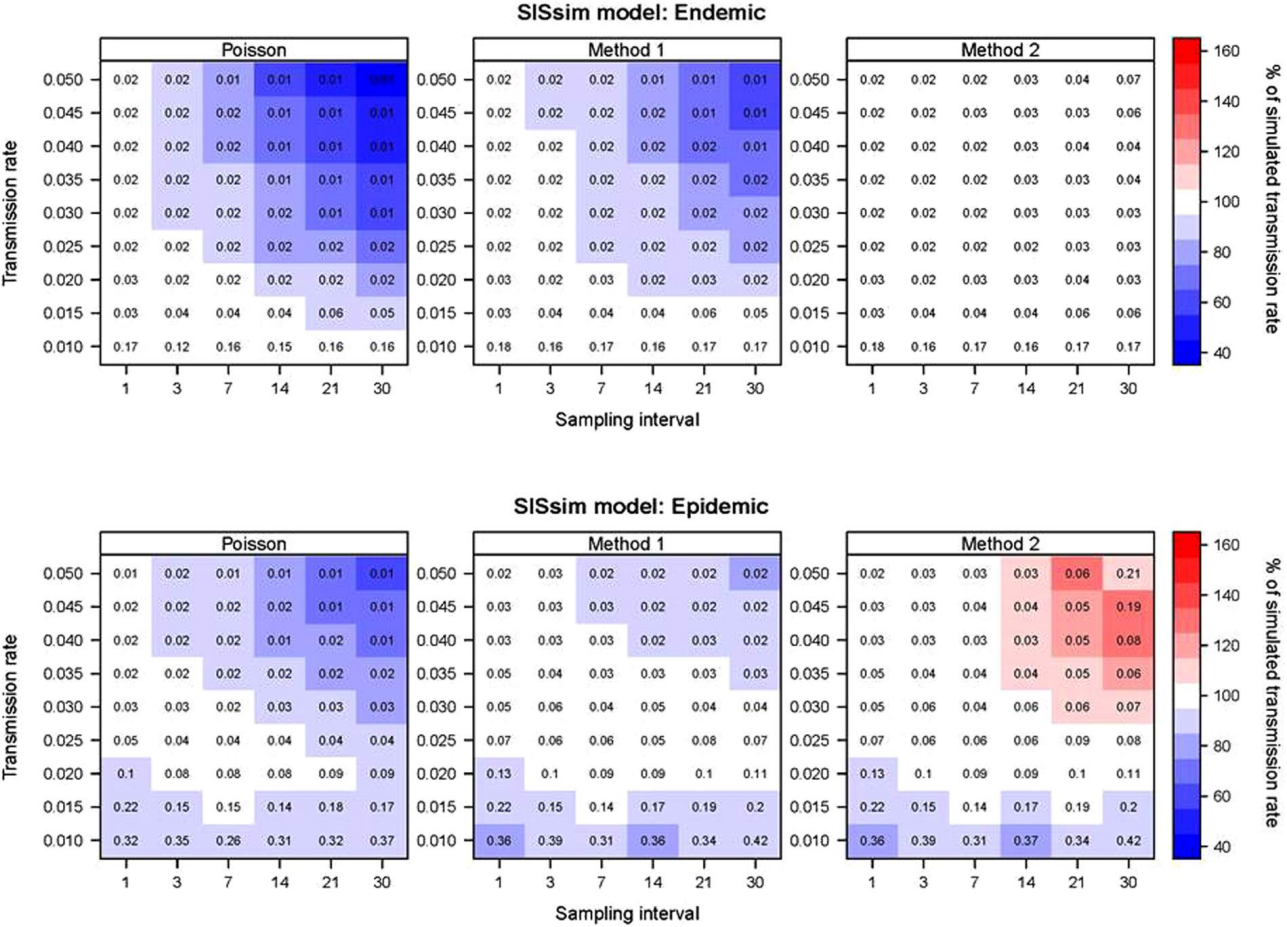
SISsim model results: Heatmaps show the estimated transmission rates as percentage of the simulated transmission rate. The endemic scenario is shown above and the epidemic below. Values in each cell show the relative standard deviation of the estimated rate divided by the simulated rate.

From the SISsim model data we see that the Poisson regression method underestimates the transmission rate, more pronounced with larger sampling intervals and higher transmission rates (Fig. 3). The Poisson regression performed better in the epidemic situation than in the endemic situation. There is almost no change in the estimate when subsampling the population down to 25%. The highest and lowest estimated rates were 106% and 58% of the actual rate in the epidemic scenario, and 100% and 41% in the endemic scenario. The relative standard deviation in the estimated rates is generally very low, with increasing variation when the test interval and transmission rates used are small (Fig. 3).

Method 1 performed better than Poisson in the epidemic scenarios. The estimated rate was between 100% and 79% in the epidemic scenario and between 100% and 55% of the simulated rate in the endemic scenario (Fig. 3). Like the Poisson model, it showed an underestimation that was more pronounced with larger sampling intervals and transmission rates. Subsampling the population had very little impact on the estimated rates, although it did increase the relative standard deviation a little (Fig. 3).

Method 2 yielded a slight overestimation with increasing sampling interval and transmission rate, ranging between 96% and 138% of the simulated rate in the epidemic scenario and between 100% and 114% in the endemic scenario (Fig. 3). Thus it performed better in the endemic scenario than the epidemic scenario. Subsampling the population affected the estimate similarly to the other two methods (Figs 1–16 in supplementary material).

The variation between results was generally much larger in the epidemic scenarios than in the endemic scenarios. In the endemic scenario, the lowest variation was in the results of the Poisson regression method. The highest variation was in the results from Method 2. In the epidemic scenario we see the same pattern where the Poisson regression method results in the lowest variation and Method 2 shows the highest variation.

### SIScom model

**Figure 4:**
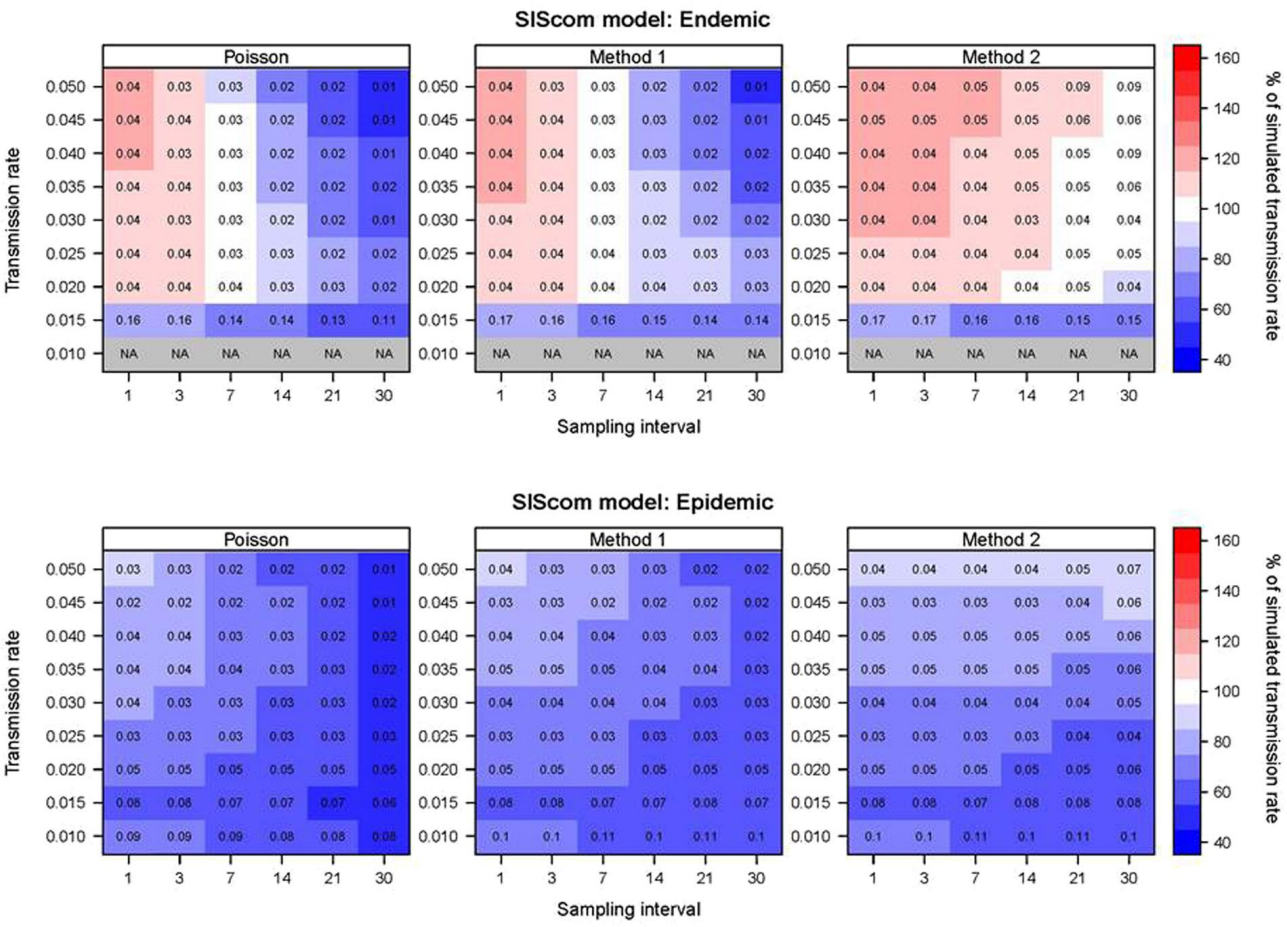
SIScom model results: Heatmaps show the estimated transmission rates as percentage of the simulated transmission rate. The endemic scenario is shown above and the epidemic below. Values in each cell show the relative standard deviation of the estimated rate divided by the simulated rate.

In the epidemic scenario, all three methods underestimated the transmission rate (Fig. 4). For Poisson and Method 1 the underestimation was worst at large sampling intervals. Method 2 performed better at long sampling intervals. Subsampling did not noticeably change the estimate, but did increased the relative standard deviation of the estimate (Figs 5–16 in supplementary material).

The endemic scenarios show both over- and underestimation. The Poisson regression and Method 1 overestimates the rate when the sampling interval is one or three days (Fig. 4). When the sampling interval was seven days, they were mostly correct. When the sampling interval is between 14 and 30 days, the Poisson method underestimated the rate. Both the overestimation at small sampling intervals and the underestimation at large sampling intervals becomes more pronounced with higher transmission rates. The relative standard deviation seems to be negatively correlated with the sampling interval. We see the same pattern for Method 1 as for the Poisson regression (Fig. 4). Using Method 2 in the endemic scenario mostly results in an overestimation of the rate, highest when the sampling interval was small and the transmission rate is high.

When the transmission rate becomes very low, at 0.010, in the endemic scenario, none of the methods were able to estimate a transmission rate because the disease had died out (Fig. 4). When the rate is 0.015, all three methods underestimate the rate.

## Discussion

We here used a dairy herd as an example of a realistic study population and showed that a good estimate of the disease transmission rate can be difficult to obtain, supporting the recent findings of Jacobs *et al*.^29^. We found considerable over- and underestimation in the results, both between the used models, and the used methods. The estimation error increased with large sampling intervals and high transmission rates. Moreover, if the transmission rate gets close to the recovery rate, the estimated transmission rate can be imprecise due to stochastic events and the probability of disease fadeout. Subsampling only reduces the precision of the estimates, although the impact was found to be very small in this study (Figs 5–16). Based on these findings, we recommend that simulation models of disease spread use transmission rates from stochastic distributions rather than fixed values or fit the transmission rate to represent different situations, to study whether conclusions may change given different transmission rate values.

The results show that the three methods have different strengths and weaknesses. Generally, Method 2 performed best in most situations (Fig. 2) In the SISsim model with the epidemic scenario Method 1 performed best in the investigated range of sampling intervals and transmission rates (Fig. 3). In the endemic scenario of the SISsim model, Method 2 was the most precise (Fig. 4). For the SIScom model in the epidemic scenario, Method 2 was found to be preferable, although it still underestimated the rate (Fig. 4). In the endemic scenario with the SIScom model, none of the methods performed optimally, but Method 2 performed overall better (Fig. 4). All methods perform similarly when the sampling interval is one day.

The transmission rate itself consists of different parameters: contact rate, susceptibility of individuals and force of infection^1^. In this study we did not separate the rate into these parameters, as this is practically impossible. Furthermore, the estimated transmission rate is biased because biological systems are practically never in perfect equilibrium, which is an underlying assumption in all the estimation methods described here. The two new methods assume that the system is close to an endemic equilibrium, while the Poisson method assumes that the number of susceptible and infected animals is stable between sampling points. Thus the estimation method itself inevitably introduces a bias to the estimate. It is more desirable to have random bias rather than systematic bias, because random bias can be cancelled out using multiple data points.

Homogenous mixing is an underlying assumption in our simulations and theoretical framework, but this is most likely not the case in real life. Other factors can also create temporal variations in disease transmission. For instance, physiological susceptibility in the individuals can be heterogeneously distributed, meaning that the most susceptible individuals can acquire the infection first, causing the transmission rate to be high in the beginning of an outbreak and then drop^1^. The infectivity could also be heterogeneously distributed within the population, adding to the complexity in real populations^21^. A further assumption of this study is that the status of individuals are only registered at sampling times (i.e. An individual that become infectious and return to susceptible within a sampling period is not recorded as a new infection).

Nielsen *et al*.^18^ mention that the time between sampling points should preferably be as short as the average infective period to optimally estimate the transmission parameters, because all transmission events are registered that way. This is true for the Poisson regression method and Method 1 used here. However, Method 2 presented here takes the possibility of transmission events between samplings into account. This means that Method 2 is not as sensitive to large sampling intervals as the two other methods. In concordance with this, we used a mean infective period of 125 days (1/0.008) in the SISsim model, and could detect the transmission rate with high precision even when sampling intervals were as large as 150 days (results not shown).

We also investigated the impact of changing the recovery rate in the SISsim model and the flare-up rate in the SIScom model. This affects the period of infection, but had a negligible effect on the estimate of transmission rate and thus the results are not shown.

The results of the SISsim and the SIScom model are not similar. The SISsim model is a simple representation of the transmission framework, from which the rate estimation methods are derived. The noise in this model only comes from stochasticity in the infection events and the subsampling. In the SIScom model, additional noise comes from the natural flow of individuals that are not sampled at all sampling points (cows that are being dried off or replaced during the study period). This represents a more realistic biological system, and the errors become more complex.

The results presented here assume that the true status of the individuals is known in both the SISsim and the SIScom model. The noise in the SIScom model comes from the natural fluctuations in a dairy herd where cows are dried off and eventually culled. However, in a more realistic setting the status of the individuals can only be known through testing, which will add the noise from the test specificity and sensitivity. We did not include this in the current study. These measures will vary with the test used, therefore we wanted to keep this out of this study, in order to focus on the precision of the used methods. This study assumes that the true transmission mode is known. If, for instance, infections were caused by mass action^1^, the estimated rate will likely be further off. However, this is beyond the scope of this study.

It is not practically possible to study the precision of the estimated transmission rate on data from a real population because this implies that the real transmission rate is known. Therefore we here used the second best solution and used a realistic dataset. The results reported here are specific to the model and data, and hence should not be generally used to make adjustments to obtained results from other studies. The best possible estimate of transmission rate will be achieved by building a simulation model closely resembling the observed case, and fit this to data.

Here we take the mean of 30 or 50 simulated datasets. In studies where the transmission rate is estimated, there is most likely only one dataset. Thus, such an estimate will not be as precise as when taking the mean of a number of datasets. Poisson and Method 1 generally have similar standard deviation in the estimates, while Method 2 typically is a factor of two higher. This means that Method 2 has a higher variance, and the estimated transmission rate can differ from the true rate. However, it is often the best method, or among the best methods in the scenarios presented here, so it is still a robust method.

If there are no new infections between sampling points, the Poisson regression analysis will give an error as the log-link returns a negative infinite value. The two new methods proposed here do not have similar problems and hence they are superior to the Poisson method when no new infections exist.

In this study, we have explored regression and analytical approaches. Another approach could be Bayesian inference^16,21,30^, or estimating time-varying transmission rates^31^. Arguably, the transmission rate does not remain constant over time^21^. For instance, Zadoks *et al*.^22^ distinguished between an endemic and an outbreak (epidemic) period of *S*. *uberis* in the studied data. Cook *et al*.^21^ used Bayesian inference to estimate transmission rates varying in time in a heterogeneous population. Thus, it can be beneficial to estimate the transmission rate for each sampling interval. This is not possible to do with the Poisson regression model, but it is contained in both Method 1 and Method 2 described here. For simplicity, we chose to use the mean of the entire study period in this study, since there will be large variations in the estimates from small systems.

The SISsim system of differential equations can be solved analytically, and in principle the epidemic curve can be fitted to this solution. However, this requires fitting of three parameters (initial value, *α*, and *β*), and when equilibrium has been reached only the ratio between *α*, and *β* is well defined. To achieve a good fit there must be some points in the epidemic part of the disease. This is often not achievable especially if the sampling interval is long. The two new methods that we have presented here are robust towards long sampling and are designed for optimal function in the endemic situation.

We also conducted negative binomial regression to estimate transmission rates in this study, but the results with this method were very similar to those with poisson regression (data not shown). Furthermore, it failed to estimate rates for many simulations with sparse transmission events, i.e. when no new infections occurred between samplings.

To conclude, our results showed that the true transmission rate is rarely obtained, and the estimated rate can be both over- and underestimated. The error in the estimate increases when sampling intervals are large and transmission rates are high. Subsampling the populations down to 25% only slightly affected the estimated rate. The presented new methods, Method 1 and Method 2, were found to be superior or equal to Poisson regression in most scenarios explored in this study.

## Electronic supplementary material

Supplementary material
