# Author Correction: Methods for estimating disease transmission rates: Evaluating the precision of Poisson regression and two novel methods

**DOI:** 10.1038/s41598-018-26491-5

**Published:** 2018-06-20

**Authors:** Carsten Kirkeby, Tariq Halasa, Maya Gussmann, Nils Toft, Kaare Græsbøll

**Affiliations:** National Veterinary Institute, Technical University of Denmark, Bülowsvej 27, DK-1870 Frederiksberg C, Denmark

Correction to: *Scientific Reports* 10.1038/s41598-017-09209-x, published online 25 August 2017

In the Supplementary Information file originally published with this Article, the references were omitted. This error has been corrected in the Supplementary Information that now accompanies the Article.

