# Supplementary material for: Methods for estimating disease transmission rates: Evaluating the precision of Poisson regression and two novel methods
Source: Sci Rep. 2017 Aug 25;7:9496. doi: 10.1038/s41598-017-09209-x (PMC5572698; doi:10.1038/s41598-017-09209-x)
Supplement: Supplementary file 1 — Supplementary material [file 41598_2017_9209_MOESM1_ESM.pdf]

# Methods for estimating disease transmission rates: Evaluating the precision of Poisson regression and two novel methods

**Carsten Kirkeby*, Tariq Halasa*, Maya Gussmann*, Nils Toft*, Kaare Græsbøll***

*Veterinary Institute, Technical University of Denmark, Bülowsvej 27, DK-1870 Frederiksberg C, Denmark

## Supplementary material

## Supplementary table

| Symbol | Name | Description | Value | Reference |
|---|---|---|---|---|
| $\beta$ | Transmission rate | Transmission (infection) rate for individuals entering subclinical or clinical state | 0.0179 | [1] |
| $1-\rho$ | Probability of clinical state | Probability of acquiring clinical infection when infected | 0.83 | [2] |
| $\rho$ | Probability of subclinical state | Probability of acquiring clinical infection when infected | 0.17 | [3] |
| $\gamma_S$ | Flare up rate | Rate of subclinical individuals going to clinical state | 0.008 | [3] |
| $\alpha_S$ | Spontaneous cure probability | Probability of spontaneous cure for subclinical individuals | 0.0064 | [1] |
| $\alpha_C$ | Recovery rate | Rate of recovery for clinical individuals that are treated going to either susceptible or subclinical individuals | 0.33 | [4] |
| $\eta$ | Recovery probability | Probability of recovery for clinical individuals that are treated | 0.40 | [4] |
| $1-\eta$ | Remission probability | Probability of remission of individuals going from clinical to subclinical state | 0.60 | [4] |

**Table 1.** Rates and probabilities used in the SIScom simulation model. All parameters are implemented in daily time steps, for all quarters. The parameters are shown in Figure 1.

# References

## 1. References


1. van den Borne, B. H., Halasa, T., van Schaik, G., Hogeveen, H. & Nielen, M. Bioeconomic modeling of lactational antimicrobial treatment of new bovine subclinical intramammary infections caused by contagious pathogens. *J. dairy science* **93**, 4034–4044 (2010).
2. Swinkels, J., Hogeveen, H. & Zadoks, R. A partial budget model to estimate economic benefits of lactational treatment of subclinical staphylococcus aureus mastitis. *J. dairy science* **88**, 4273–4287 (2005).
3. Halasa, T., Nielen, M., Huirne, R. & Hogeveen, H. Stochastic bio-economic model of bovine intramammary infection. *Livest. Sci.* **124**, 295–305 (2009).
4. Steeneveld, W., van Werven, T., Barkema, H. W. & Hogeveen, H. Cow-specific treatment of clinical mastitis: An economic approach. *J. Dairy Sci.* **94**, 174–188 (2011).


## Supplementary figures

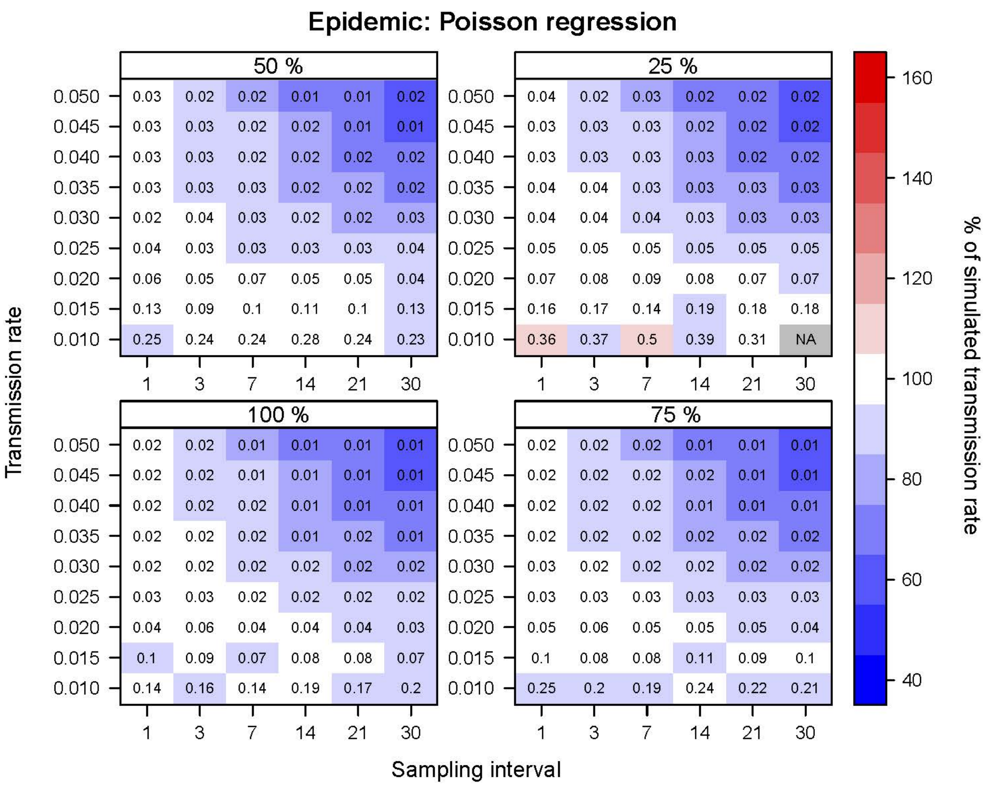


**Figure 5.** SISsim model, epidemic situation, poisson regression: Heatmaps showing the estimated transmission rates as percentage of the simulated transmission rate. The estimates are shown for four subsampling levels: 100%, 75%, 50% and 25%. Values in each cell show the relative standard deviation of the estimated rate divided by the simulated rate.

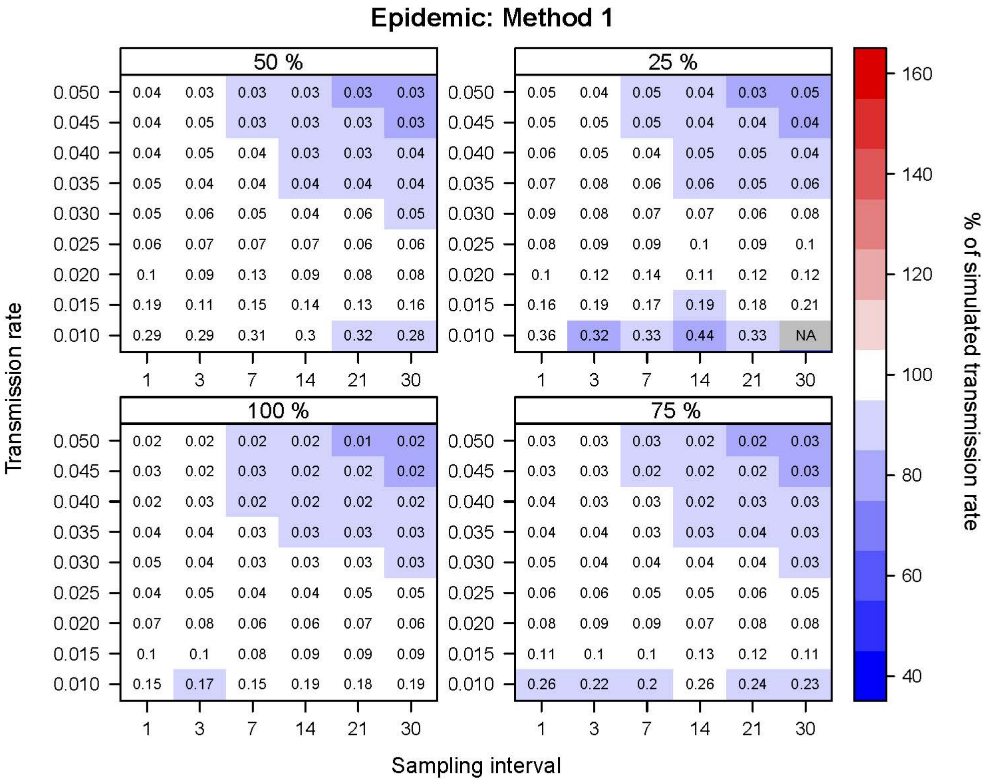


**Figure 6.** SISsim model, epidemic situation, Method 1: Heatmaps showing the estimated transmission rates as percentage of the simulated transmission rate. The estimates are shown for four subsampling levels: 100%, 75%, 50% and 25%. Values in each cell show the relative standard deviation of the estimated rate divided by the simulated rate.

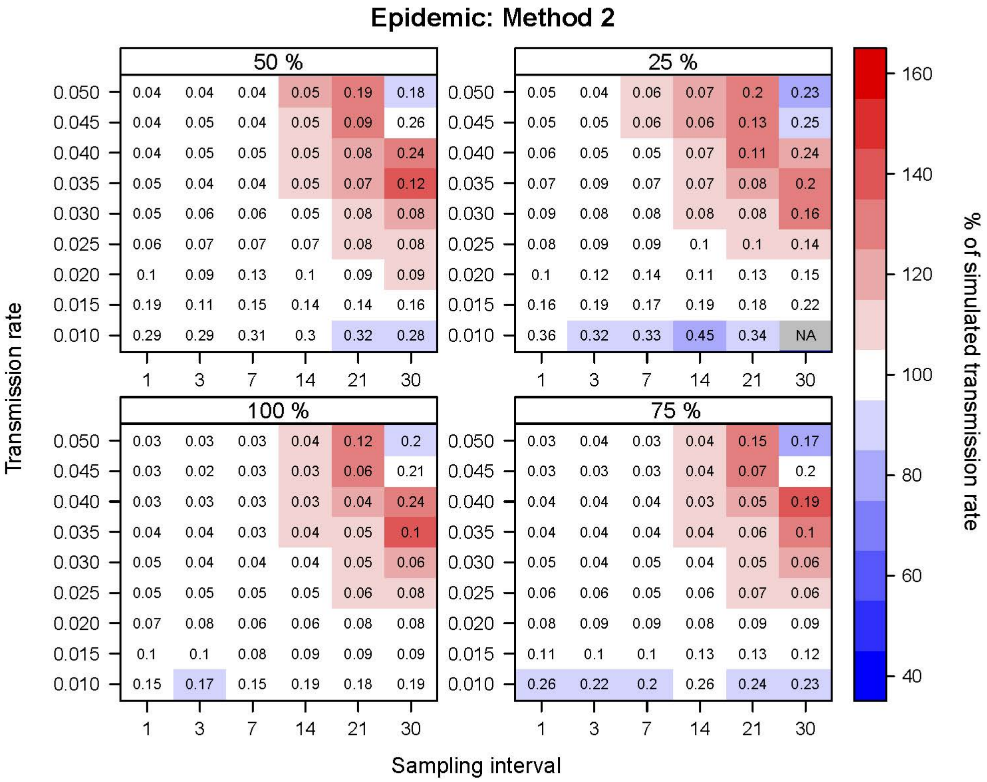


**Figure 7.** SISsim model, epidemic situation, Method 2: Heatmaps showing the estimated transmission rates as percentage of the simulated transmission rate. The estimates are shown for four subsampling levels: 100%, 75%, 50% and 25%. Values in each cell show the relative standard deviation of the estimated rate divided by the simulated rate.

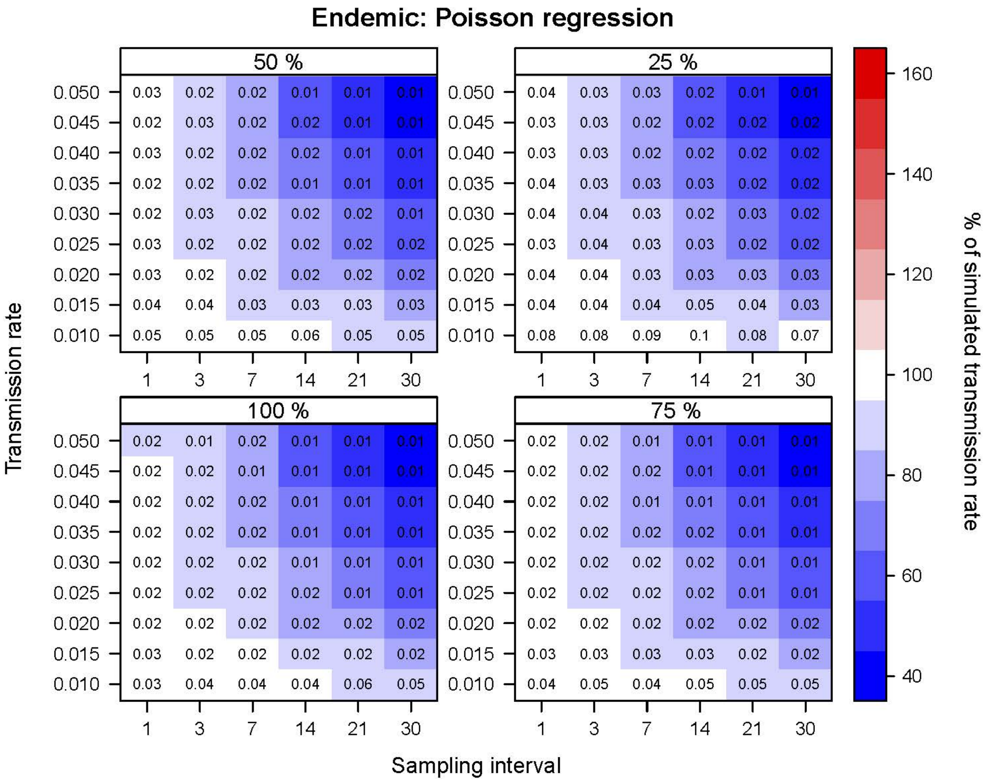


**Figure 8.** SISsim model, endemic situation, Poisson regression: Heatmaps showing the estimated transmission rates as percentage of the simulated transmission rate. The estimates are shown for four subsampling levels: 100%, 75%, 50% and 25%. Values in each cell show the relative standard deviation of the estimated rate divided by the simulated rate.

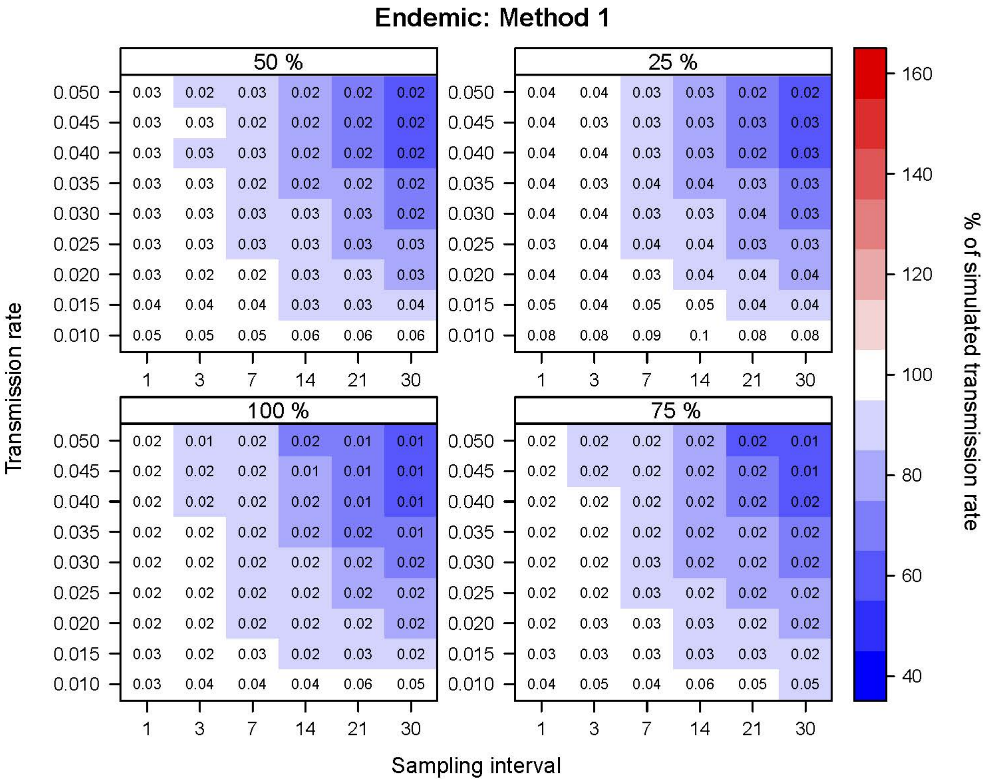


**Figure 9.** SISsim model, endemic situation, Method 1: Heatmaps showing the estimated transmission rates as percentage of the simulated transmission rate. The estimates are shown for four subsampling levels: 100%, 75%, 50% and 25%. Values in each cell show the relative standard deviation of the estimated rate divided by the simulated rate.

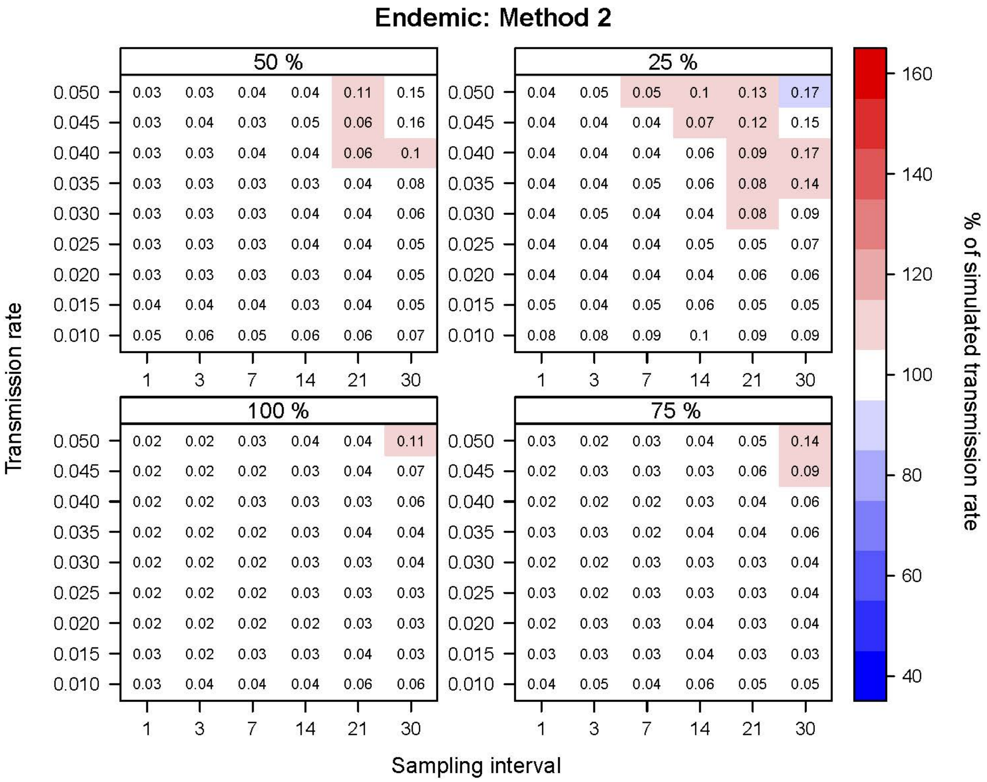


**Figure 10.** SISsim model, endemic situation, Method 2: Heatmaps showing the estimated transmission rates as percentage of the simulated transmission rate. The estimates are shown for four subsampling levels: 100%, 75%, 50% and 25%. Values in each cell show the relative standard deviation of the estimated rate divided by the simulated rate.

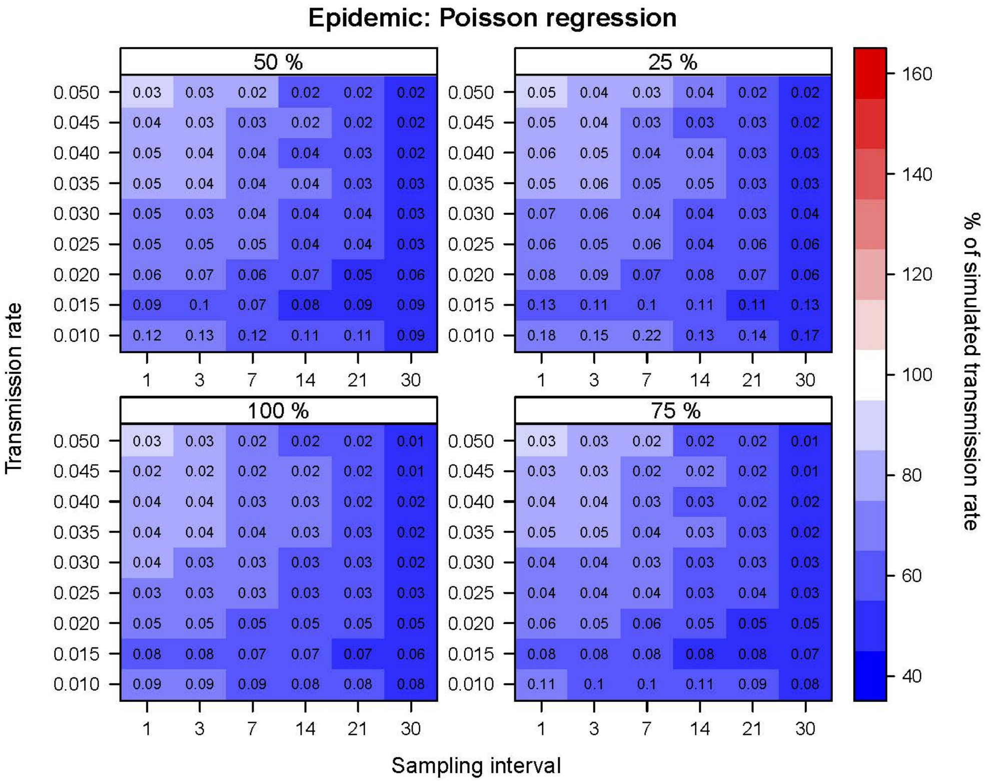


**Figure 11.** SIScom model, epidemic situation, poisson regression: Heatmaps showing the estimated transmission rates as percentage of the simulated transmission rate. The estimates are shown for four subsampling levels: 100%, 75%, 50% and 25%. Values in each cell show the relative standard deviation of the estimated rate divided by the simulated rate.

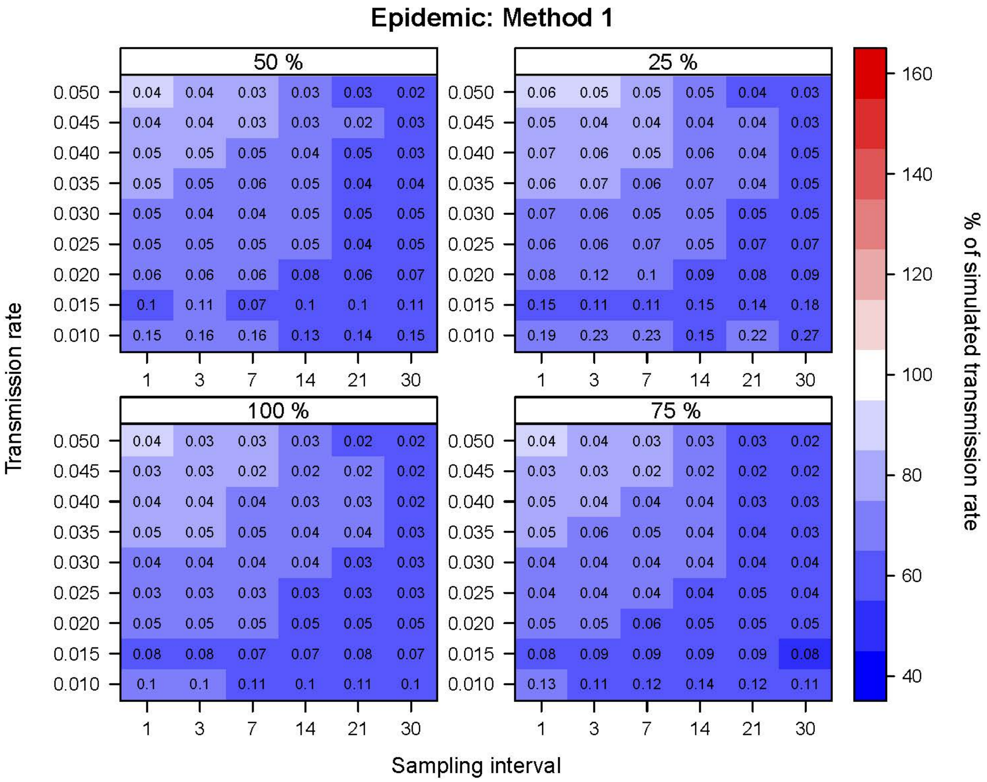


**Figure 12.** SIScom model, epidemic situation, Method 1: Heatmaps showing the estimated transmission rates as percentage of the simulated transmission rate. The estimates are shown for four subsampling levels: 100%, 75%, 50% and 25%. Values in each cell show the relative standard deviation of the estimated rate divided by the simulated rate.

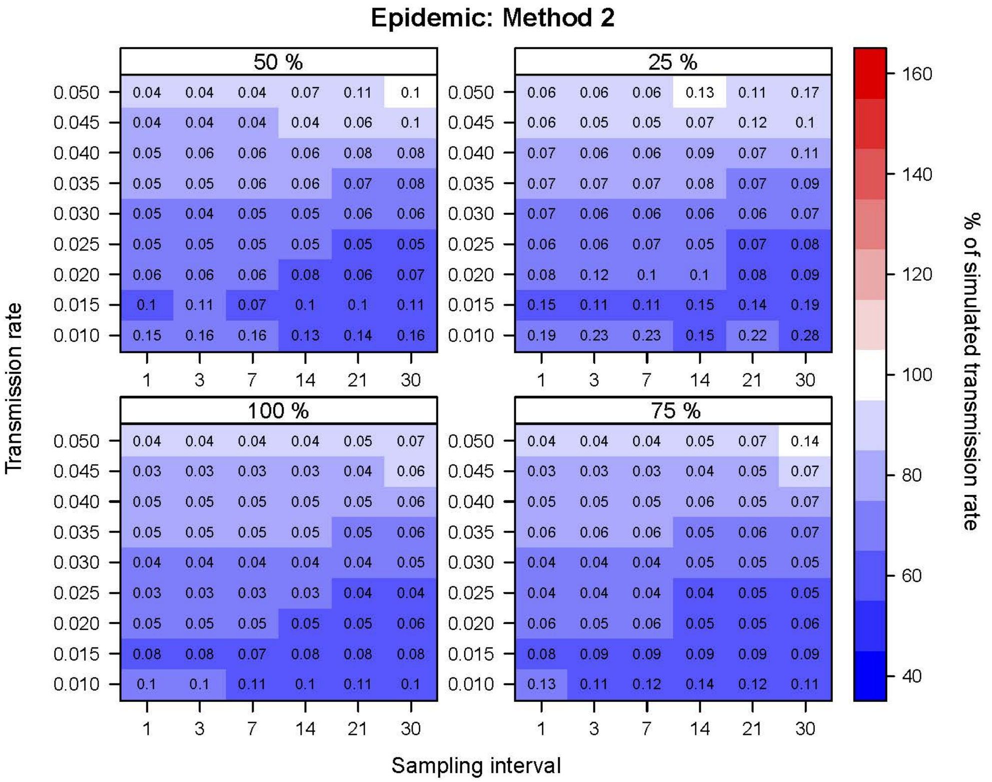


**Figure 13.** SIScom model, epidemic situation, Method 2: Heatmaps showing the estimated transmission rates as percentage of the simulated transmission rate. The estimates are shown for four subsampling levels: 100%, 75%, 50% and 25%. Values in each cell show the relative standard deviation of the estimated rate divided by the simulated rate.

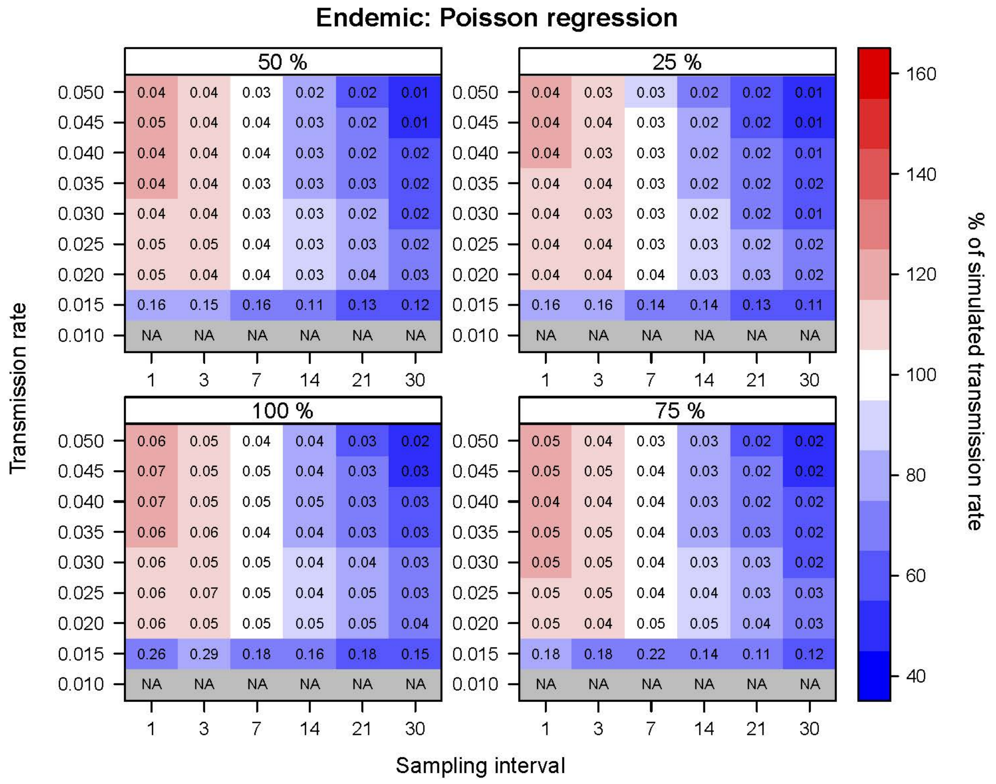


**Figure 14.** SIScom model, endemic situation, poisson regression: Heatmaps showing the estimated transmission rates as percentage of the simulated transmission rate. The estimates are shown for four subsampling levels: 100%, 75%, 50% and 25%. Values in each cell show the relative standard deviation of the estimated rate divided by the simulated rate.

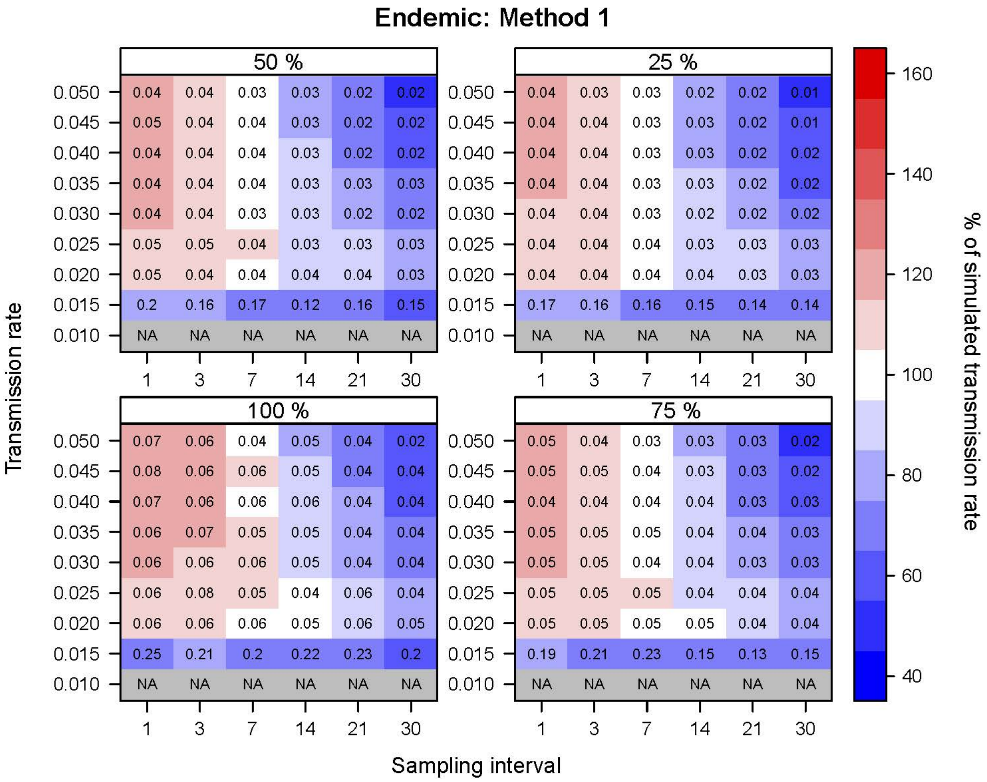


**Figure 15.** SIScom model, endemic situation, Method 1: Heatmaps showing the estimated transmission rates as percentage of the simulated transmission rate. The estimates are shown for four subsampling levels: 100%, 75%, 50% and 25%. Values in each cell show the relative standard deviation of the estimated rate divided by the simulated rate.

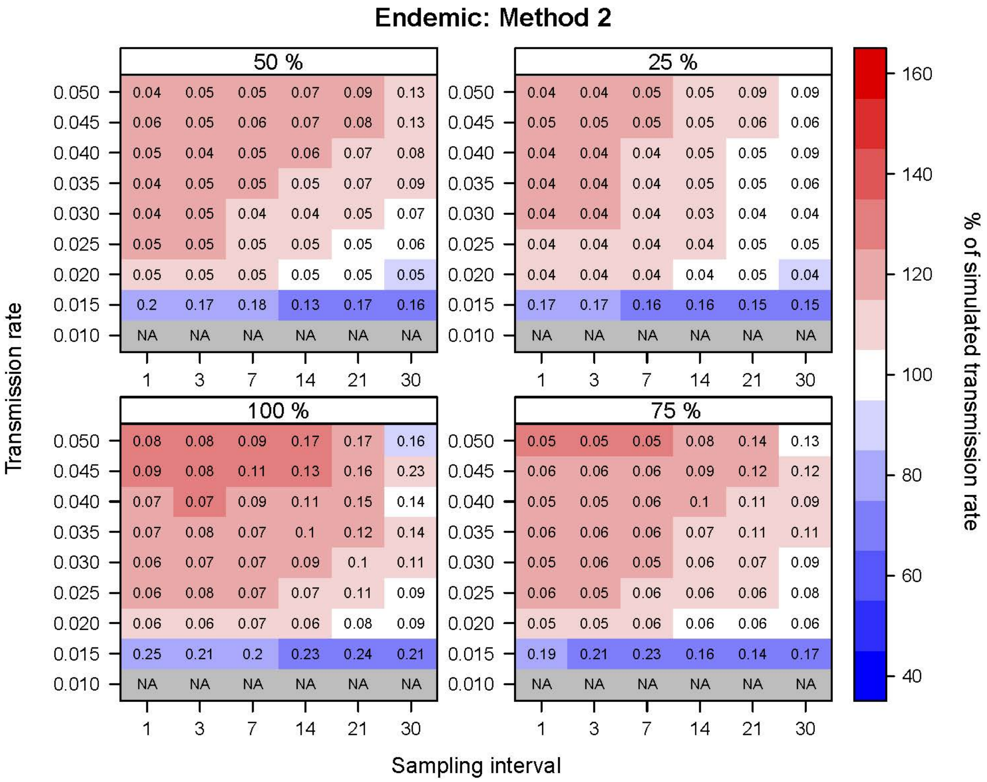


**Figure 16.** SIScom model, endemic situation, Method 2: Heatmaps showing the estimated transmission rates as percentage of the simulated transmission rate. The estimates are shown for four subsampling levels: 100%, 75%, 50% and 25%. Values in each cell show the relative standard deviation of the estimated rate divided by the simulated rate.
